# One Size Fits All? Not in *In Vivo* Modeling of Tuberculosis Chemotherapeutics

**DOI:** 10.3389/fcimb.2021.613149

**Published:** 2021-03-16

**Authors:** Hee-Jeong Yang, Decheng Wang, Xin Wen, Danielle M. Weiner, Laura E. Via

**Affiliations:** ^1^ Tuberculosis Research Section, Laboratory of Clinical Immunology and Microbiology, Division of Intramural Research (DIR), National Institute of Allergy and Infectious Disease (NIAID), National Institutes of Health (NIH), Bethesda, MD, United States; ^2^ Medical College, China Three Gorges University, Yichang, China; ^3^ Institute of Infection and Inflammation, China Three Gorges University, Yichang, China; ^4^ Tuberculosis Imaging Program, DIR, NIAID, NIH, Bethesda, MD, United States; ^5^ Institute of Infectious Disease and Molecular Medicine, University of Cape Town, Cape Town, South Africa

**Keywords:** *Mycobacterium tuberculosis*, rabbit, guinea pig, non-human primate, drug development, zebrafish, chemotherapy, animal model

## Abstract

Tuberculosis (TB) remains a global health problem despite almost universal efforts to provide patients with highly effective chemotherapy, in part, because many infected individuals are not diagnosed and treated, others do not complete treatment, and a small proportion harbor *Mycobacterium tuberculosis (Mtb*) strains that have become resistant to drugs in the standard regimen. Development and approval of new drugs for TB have accelerated in the last 10 years, but more drugs are needed due to both *Mtb*’s development of resistance and the desire to shorten therapy to 4 months or less. The drug development process needs predictive animal models that recapitulate the complex pathology and bacterial burden distribution of human disease. The human host response to pulmonary infection with *Mtb* is granulomatous inflammation usually resulting in contained lesions and limited bacterial replication. In those who develop progressive or active disease, regions of necrosis and cavitation can develop leading to lasting lung damage and possible death. This review describes the major vertebrate animal models used in evaluating compound activity against *Mtb* and the disease presentation that develops. Each of the models, including the zebrafish, various mice, guinea pigs, rabbits, and non-human primates provides data on number of *Mtb* bacteria and pathology resolution. The models where individual lesions can be dissected from the tissue or sampled can also provide data on lesion-specific bacterial loads and lesion-specific drug concentrations. With the inclusion of medical imaging, a compound’s effect on resolution of pathology within individual lesions and animals can also be determined over time. Incorporation of measurement of drug exposure and drug distribution within animals and their tissues is important for choosing the best compounds to push toward the clinic and to the development of better regimens. We review the practical aspects of each model and the advantages and limitations of each in order to promote choosing a rational combination of them for a compound’s development.

## Introduction

The WHO global tuberculosis report estimated there were 10 million new active tuberculosis (TB) cases and 1.4 million TB-associated deaths in 2019 (Global Tuberculosis Report, 2020). Additionally, 5% of TB cases were rifampicin-resistant with 78% of those being multidrug-resistant (MDR) posing severe challenges for public health agencies providing treatment. Although there are different estimates of the pool of persons currently living with latent TB infection (LTBI), these persons need to be identified, assessed for risk of developing active disease, and those at risk treated if we are to eliminate Tuberculosis by 2050 (Esmail et al., 2014; Houben and Dodd, 2016; Kaplan, 2020). In humans, the vast majority of TB is caused by *Mycobacterium tuberculosis* (*Mtb*) but may also be caused by another member of the *Mtb* complex (*Mtbc*), *Mycobacterium africanum. Mycobacterium bovis* (*Mb*) and other members of the animal-adapted *Mtbc* typically cause tuberculosis in other mammals (Brites and Gagneux, 2017). Within *Mtb* there are groups, or lineages, of strains that are somewhat geographically restricted or “ancient” that retain a chromosomal region called Mtb-specifc deletion 1 (TbD1) that was lost from the progenitor of the widespread or modern lineages (Brites and Gagneux, 2017; Bottai et al., 2020). The effect of this deletion was to give modern *Mtb* greater tolerance to oxidative stress and hypoxia (Bottai et al., 2020) which translated to greater virulence in infection models with human-like necrotic lesions. The most common lab strains used for compound screening and animal experiments are derived from the modern lineages which include the Beijing, CAS/Dehli, and Euro-American strain families, suggesting that we may be missing some heterogeneity that could be important to recognize.

*Mtb* infections are notoriously difficult to cure, in part because of *Mtb’s* waxy mycobacterial cell wall, slow growth rate, and natural drug resistance, but also because of its ability to develop phenotypic drug tolerance and persist for long periods in the host before proliferating and causing symptomatic disease (Brennan, 2003; Barry et al., 2009; Aldridge et al., 2014; Gordon and Parish, 2018). After decades of little activity, numerous compounds, new drug candidates, and possible combinations are being pushed forward for assessment: the question is how to best evaluate the compounds that advance into *in vivo* testing (https://www.newtbdrugs.org) (Libardo et al., 2018; Tornheim and Dooley, 2019; Huszár et al., 2020). Additional factors to be considered are the complex pathology characteristics of human disease and that successful treatment may rely both on compound distribution into regions of disease and the differential abundance and susceptibility of the bacteria in these diverse regions (Kaplan et al., 2003; Prideaux et al., 2015; Hunter, 2016; Hunter, 2018; Strydom et al., 2019; Sarathy and Dartois, 2020). The characteristics of useful *in vivo* models for evaluating new compounds include: development of relevant disease through a low dose *Mycobacterium* exposure; development of a persistent infection with both intracellular and extracellular bacteria; development of heterogenous lesion pathology, including granulomas and pneumonia with regions of necrosis with relatively low bacterial loads and cavities with highly heterogenous bacterial loads (Kaplan et al., 2003; Hunter, 2016); as well as being amenable to methods for assessing the state of the infection *in situ*. Also needed are methods for assessing compound exposure both systemically and locally at the site of disease in as small an animal as practical to minimize compound requirements. This review focuses on the more common vertebrate animal models of tuberculosis used for compound assessment and will address the following: their general characteristics, with elaboration on the biological contributions of each towards assessment of current drugs and compounds; the advantages, practicalities, and limitations of each; and suggestions for combining the models, as no one model mimics the time course and complex pathology of human tuberculosis.

## Zebrafish are an Effective Platform for Anti-Tuberculosis Compound Screening

**Figure 1:**
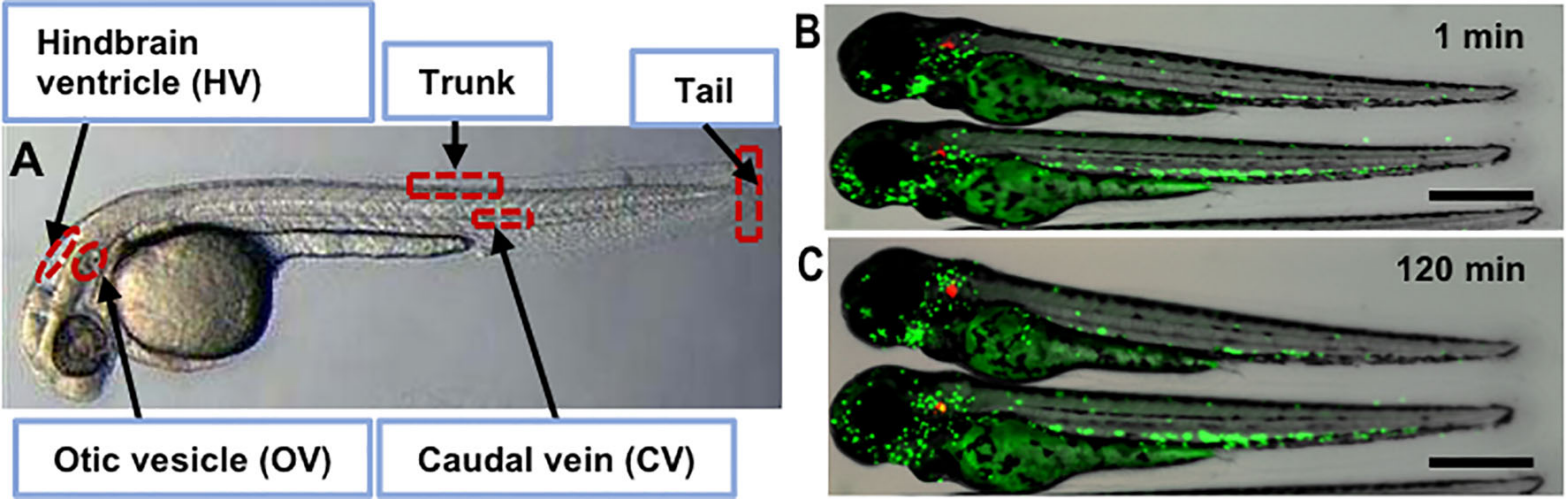
Zebrafish infection sites and zebrafish larva infected with *M. marinum*. **(A)** Bright field image of sites used for microinjection of the pathogen including otic vesicle, hindbrain, caudal vein, tail and trunk. Fluorescence and bright field overlay image of transgenic zebrafish with Dendra2-expressing neutrophils (green) after injection of tdTomato-expressing *Mm* (red) into the OV site after 1 min **(B)** and 120 min **(C)** showing neutrophil migration. Scale bar equals 500 μm.

Zebrafish (*Danio rerio*) were originally used to study the stages of vertebrate development, in part because the larval body was transparent and thus conducive for live imaging (Kimmel et al., 1995). In addition, the 2–4 cm fish breed quickly (100–200 eggs/week), share important similarities with the mammalian immune system, and are genetically tractable so they can be engineered to produce fluorescent protein markers in specific cell lineages (**Figure 1**) (Renshaw and Trede, 2012; Henry et al., 2013; Bernut et al., 2016; Hsu et al., 2017). The initial interest in zebrafish as a model for tuberculosis was twofold: they are a natural host for *Mycobacterium marinum* (*Mm*), a close relative and evolutional homolog of *Mtb*, without requiring biological safety level 3 (ABSL-3) containment; and the model allows the genetics of tubercular disease to be studied through mutations in both host and pathogen (Prouty et al., 2003; Berg and Ramakrishnan, 2012; Belon et al., 2014; Luukinen et al., 2018; Tükenmez et al., 2019). Both adult and larval zebrafish develop granulomatous lesions (Cosma et al., 2004; Volkman et al., 2004; Swaim et al., 2006; Davis and Ramakrishnan, 2009; Volkman et al., 2010; Franzblau et al., 2012; Oehlers et al., 2015; Cronan et al., 2016; Fenaroli et al., 2018). *Mm* initiates primary granuloma formation by inducing nearby epithelial cells to release matrix metalloproteinase-9, thus recruiting macrophages, promoting granuloma maturation and bacterial replication, much like *Mtb* (Volkman et al., 2010). Macrophage phagocytosis of *Mm* transforms them into foamy macrophages, similar to *Mtb* in mammalian species, thus sustaining persistent bacteria (Peyron et al., 2008; Johansen et al., 2018). The development of disease is *Mm* dose dependent: in low-dose infection of adults [~24 to 30 colony forming units (CFU)], *Mm* can induce a persistent subclinical (or latent) state that can be reactivated by *γ*-irradiation to temporarily deplete immune cells; in high-dose infection of adults (~1,800 to 2,500 CFU), *Mm* can induce a more active state (Parikka et al., 2012). Another approach to studying persistent infection was developed by creating a *Mm* mutant lacking functional resuscitation-promoting factors AB (Commandeur et al., 2020). This mutant developed a persistent phenotype in zebrafish embryos if the bacteria were nutrient-starved before inoculation. The model was tolerant to ethambutol (EMB) and was proposed as a screening tool for compounds active against asymptomatic infections. According to the cumulative literature, different inoculation routes of *Mm* can be used: adult zebrafish are typically infected *via* intraperitoneal (ip) or intramuscular (im) injection, whereas embryonic-larval infection is typically *via* caudal vein (cv) injection, although there are a number of local inoculation routes possible (**Figure 1A**). Following infection with fluorescent protein-expressing *Mm*, host pathogen interactions can be directly observed with time-lapse photomicrography of transgenic fish larvae with fluorescent protein-labeled cells, for example neutrophils, like those shown in **Figures 1B, C** and discussed in Belon et al. (2014).

The zebrafish model is utilized for high-throughput drug discovery research in both embryonic–larval developmental stages as well as the adult developmental stages (**Table 1**). Using the embryonic–larval stages allows for drug uptake through the skin and real-time readouts of drug efficacy on a large scale due to their transparency that lasts several days into larval development (Meijer and Spaink, 2011). Assessment of drug efficacy can be performed by automated fluorescence microscopy or automated plate fluorimetry, making a 5-day or more experimental time course possible. To demonstrate this, four standard anti-TB drugs were shown to inhibit *Mm* growth inside the larvae over 4 days (Takaki et al., 2013). Since a single pair of fish produces hundreds of offspring, large numbers of embryos can be dedicated to *in vivo* minimum inhibitory concentration (MIC) profiling for compound libraries, as is done in cell culture models. In addition, zebrafish embryos have been used to evaluate developmental toxicity of compounds, such as two dithiocarbamates, previously found to inhibit *β*-carbonic anhydrase 3 (b-CA3) of *Mtb in vitro* (Aspatwar et al., 2017). The parameters evaluated for toxicity in embryos (N = 30 embryos per inhibitor) included: survival, movement, yolk sack absorption, hatching, heartbeat, body shape, and edema. While one compound had serious adverse effects on embryo development, the second compound, Fc14-584B, had no serious adverse effects and showed activity against *Mm* measured by fluorescence (Aspatwar et al., 2017). Isoniazid (INH), a first-line anti-tuberculosis drug, has shown hepatotoxicity in zebrafish larvae, demonstrated *via* activation of reactive oxygen species-mediated endoplasmic reticulum stress, apoptosis, and upregulation of the Nrf2 pathway (Jia et al., 2019). Notably, vascular endothelial growth factor (VEGF) mediated angiogenesis was necessary for *Mm*-induced granuloma formation in larval zebrafish (Oehlers et al., 2015). Blocking VEGF activity was demonstrated as a host-directed therapy (HDT) for tuberculosis that reduced bacterial replication, which was also observed in *Mtb* strain HN878-infected rabbit model to reduce hypoxia and dissemination from the lung in mice (Datta et al., 2015; Polena et al., 2016). An adult zebrafish model of *Mm*-infection and subsequent chemotherapy was also developed; the anti-mycobacterial drugs INH, rifampicin (RIF), moxifloxacin (MOX), EMB, and INH + RIF were administered orally, and the effects were evaluated, concluding all drugs tested in this model were effective (Sridevi et al., 2014). Taken together, *Mm*-infected zebrafish larvae coupled with improved automated imaging acquisition systems provide a reproducible and efficient model for: 1) evaluation of drug safety and toxicity; 2) rapid-screening of anti-tuberculosis drug effectiveness; 3) identification of potential HDT approaches. A limitation of screening in this model is that *Mm* antibiotic susceptibility is not identical to that of *Mtb*, so there is some risk of missing active compounds, but this *in vivo* screen may pick up other compounds that are only active in the presence of the host (Dalton et al., 2017; Huang et al., 2018).

**Table 1:** Vertebrate animal models typically used for tuberculosis drug testing, relevant characteristics, types of studies they contribute to in elucidating the human disease state.

Model strains(Mycobacterium species used)	Infection weight or (age)^a^; number of animals per group	Types of lesions typically observed	Special features & Typical chemotherapyuses	Main References
Zebrafish *(Mm* natural pathogen*)* *(M. abscessus)*	**Larva** (2 days) ~15 per group **Adult** (>3 month) ~15 per group	Non-necrotizing/cellular lesions, Cellular and necrotic granulomas	BSL-2 Model Compound screening, PK/PD, Efficacy, Evaluation of HDT, LTBI with γ-irradiation reactivation	(Prouty et al., 2003; Cosma et al., 2006; Davis and Ramakrishnan, 2009; Parikka et al., 2012; Sridevi et al., 2014; Luukinen et al., 2018) (Bernut et al., 2016)
Inbred Mouse C57BL/6, BALB/c, (*Mtb* H37Rv and other strains)	18–25 g (5–6 week-old) 4–5 per group	Non-necrotizing, cellular lesions	Initial PK/PD, Reticulocyte toxicity, Efficacy in acute and chronic infection, LTBI, Relapse rates	(Rhoades et al., 1997; Scanga et al., 1999; Witt et al., 2008; Gill et al., 2009; Rullas et al., 2010)
C3HeB/FeJ (*Mtb* HN878 is typical)	20–50 g (6–8 week-old) >4 per group	Full spectrum of cellular, granulocytic pneumonia, caseous necrotic, and cavitary lesions	Efficacy in acute and chronic infection, Relapse rates, LTBI, drug penetration	(Irwin et al., 2014; Dutta et al., 2014; Irwin et al., 2015; Lanoix et al., 2015; Irwin et al., 2016; Saini et al., 2019)
Humanized NOD/SCID *γ*c (*M*tb H37Rv)	~21 g (12 weeks after engraftment) 4–5 per group	Cellular lesions, caseous necrotic lesions, coalescing parenchyma.	Efficacy in acute and chronic infection,	(Calderon et al., 2013; Arrey et al., 2019)
Guinea Pig Dunkin-Harley strain (*Mtb H37Rv* and clinical strains)	300–1,000 g (>3 months) 4–6 per group -outbred	Cellular lesions, granulocytic pneumonia, caseous necrotic lesions,	Efficacy in acute and chronic disease, HDT, modeling co-morbidities	(Lenaerts et al., 2007; Clark et al., 2011; Clark et al., 2014; Chen et al., 2017)
Rabbit New Zealand White strain (*M. bovis*, *Mtb* strain *HN878, H37Rv* or *CDC1551*)	3–5 kg (> 5 months) 4–6 per group -outbred	Full spectrum of cellular, granulomatous, granulocytic pneumonia, cavitary and frequent spontaneously healing lesions	PK/PD, Efficacy in acute and chronic disease, drug penetration, TBM, bone, skin models, HDT, LTBI (H37Rv or CDC1551)	(Manabe et al., 2008; Subbian et al., 2012; Via et al., 2012; Subbian et al., 2016; Tsenova et al., 2020)
Common Marmoset (*Mtb* strain *CDC1551)*	250–450 g (>1.8 years) 4–5 per group -outbred	Full spectrum of cellular, granulomatous, granulocytic pneumonia, cavitary and rare spontaneously healing lesions	PK/PD, Efficacy in acute disease, drug penetration, HDT	(Via et al., 2013; Via et al., 2015; Cadena et al., 2016)
Rhesus and Cynomolgus Macaques (*Mtb* strain *Erdmann or H37Rv)*	3–9 kg Infants to adult (>3 years) Group size variable-outbred	Full spectrum of cellular, granulomatous, granulocytic pneumonia, cavitary and frequent spontaneously healing lesions	PK/PD, LTBI, Efficacy in acute disease, HDT, Pediatric TB, SIV/TB coinfection	(Lin et al., 2013; Rayner et al., 2013; Coleman et al., 2014a; Scanga and Flynn, 2014; Sharpe et al., 2017)

a Laboratory Animal Medicine (Fox et al., 2015).

## Mice: The Workhorses for Initial Compound Characterization

Although many animal models are employed in TB research, mice are the most commonly used animal model for tuberculosis drug development (**Table 1**) (Orme and Ordway, 2016; Nuermberger, 2017). Mice are usually infected *via* the respiratory tract currently (aerosol, intranasal, or intratracheal route) although intravenous injection was used in many early, important studies (McCune et al., 1956; McCune and Tompsett, 1956; McCune et al., 1966a; McCune et al., 1966b; Lenaerts et al., 2008). In most cases, *Mtb* replicates exponentially in the lung for the first 2 to 4 weeks and remains stable afterward, entering a chronic phase of infection (Gill et al., 2009). Although the outcome of the infection varies depending on the *Mtb* dose, the virulence of *Mtb* strain, and the susceptibility of the mouse strain (Franzblau et al., 2012; Nuermberger, 2017), a high dose of aerosol exposure (implantation of > ~5 × 10^3^ CFU in the lung) of *Mtb* results in overwhelming infection and death within 4 to 6 weeks post-infection (PI), whereas, a low-dose infection (implantation of ~10 to 500 CFU in the lung) in TB resistant mice, such as BALB/c and C57BL/6, results in a more controlled infection by the host’s immune system, developing limited lung pathology. This may be a potential limitation, particularly in drug development, because it leads to a misinterpretation of therapeutic efficacy (Dhillon et al., 1998; Mitchison and Chang, 2009; Driver et al., 2012; Lin et al., 2012; Williams et al., 2012; Irwin et al., 2014; Lanoix et al., 2015; Irwin et al., 2016). Nevertheless, many advances in drug and regimen development for TB have been guided by results in mice, such as the validation of short-course chemotherapy by addition of pyrazinamide (PZA) and RIF (Grosset, 1978; Mitchison, 1980). Detailed mouse strains and disease models used in TB drug development will be described in the following section.

### Inbred Models

Inbred mice are genetically homogeneous and have little variation or heterogeneity because they were created by mating brother/sister pairs over more than 20 generations, leading to ~99% homozygous loci within a population (Green, 1975; Festing, 1992). This might reduce experimental variability and increase study reproducibility; however, susceptibility to *Mtb* infection and treatment outcomes differ within inbred mouse strains (Lecoeur et al., 1989; Medina and North, 1998). Inbred strains CBA, DBA/s, C3H, 129/SvJ are highly permissive to *Mtb* infection, whereas BALB/c and C57BL/6 are the most resistant (Medina and North, 1998; Mitsos et al., 2000). The most widely used inbred mice are C57BL/6 and BALB/c. C57BL/6 mice infected with a moderate dose of *Mtb* (~1,000 CFU implantation in the lung) *via* aerosol develop a chronic form of pulmonary disease that progresses in several stages: expansion of the infection for the first few weeks (namely acute TB), followed by a long term chronic disease lasting at least 6 months, and finally progression to moribundity (Rhoades et al., 1997). Early bactericidal activity of INH against *Mtb* infection has been validated in acute TB models (Nikonenko et al., 2004; Rullas et al., 2010), whereas the sterilizing efficacy of RIF and PZA in the chronic model of TB proved to be more effective against slow or non-replicating bacilli (Nuermberger, 2011). These mice, along with Swiss outbred mice, have been used in the relapsing mouse model for determining bacteria sterilizing activity for a number or regimens (Andries et al., 2010; Nuermberger, 2011; Li S.Y. et al., 2017). More recently, a modification of the Cornell model that uses *Mtb* culture-filtrate to provide resuscitation-promoting factors (RPFs) to enable growth of persistent *Mtb* from the tissues has been developed (Liu et al., 2018). This study compared standard four-drug therapy with MOX substituted for INH in one arm and for EMB in a third arm; while the MOX for the INH arm was superior in speed of bactericidal clearance and relapse rates, none of the arms cleared RPF-dependent persistent *Mtb*. Using this same modification, a BDQ-containing regimen was shown to eradicate persistent *Mtb* in 8 weeks of treatment in comparison to RIF containing regimens that required 14 weeks (Hu et al., 2019). C57BL/6 mice fail to induce caseous necrotic pathology, including cavitary pathology, at any stage of infection; they develop uniform cellular lesions composed mainly of epithelioid cells mixed with lymphocyte aggregates in both acute and chronic stages of infection (Rhoades et al., 1997; Actor et al., 1999). The absence of a full range of characteristic lung pathology observed in human TB and their low genetic diversity are potential limitations of these mice in the realistic evaluation of drug efficacy achieved in humans (Helke et al., 2006; Hunter et al., 2007; Basaraba, 2008; Harper et al., 2012). These shortcomings have motivated the development of mouse populations such as C3HeB/FeJ, Collaborative Cross (CC), and the humanized mouse models.

**Figure 2:**
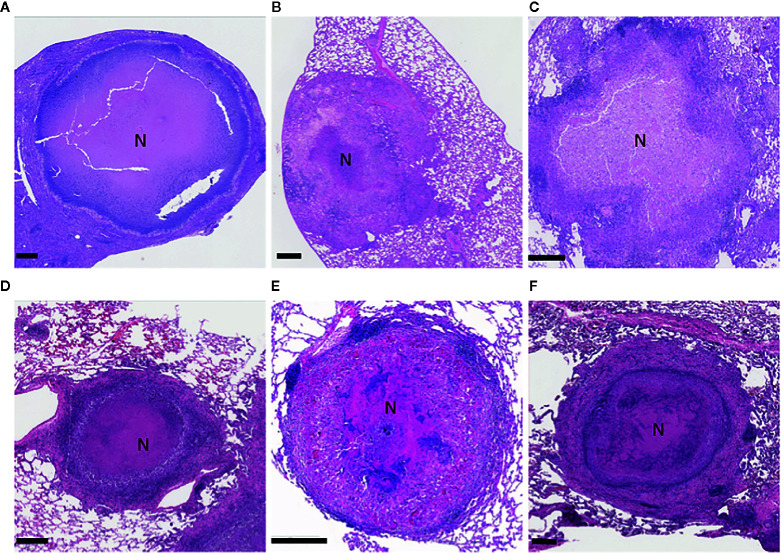
Illustration of granulomatous, necrotizing lesions in the lungs of the model animals experimentally infected with *Mycobacterium tuberculosis*. C3HeB/FeJ mouse **(A)**; guinea pig **(B)**; NZW rabbit **(C)**; marmoset **(D)**; rhesus macaque **(E)**; and a comparative human lesion **(F)**. Formalin-fixed, paraffin-embedded lung sections were stained with hematoxylin and eosin. The region of central necrosis in the lesions is marked with an N. Scale bars indicate 400 μm.

The C3HeB/FeJ mice (JAX stock #000658) are a substrain of the C3H mice that carry a recessive allele in the *sst 1* (super susceptibility to tuberculosis-1) locus that regulates macrophage innate response (Kramnik et al., 2000; Pan et al., 2005; Pichugin et al., 2009; Kramnik and Beamer, 2016). This mouse model overcomes some limitations observed in conventional laboratory mice in that they develop well defined heterogenous granulomas, as is seen in human TB (Pan et al., 2005; Driver et al., 2012; Harper et al., 2012; Rosenthal et al., 2012; Irwin et al., 2015; Lanoix et al., 2015). After infecting mice with low-dose *Mtb via* aerosol, C3HeB/FeJ mice develop three morphologically distinct types of lung lesions at about 5 weeks post infection. Irwin et al. described these three types of lesions based on cellular composition, degree of immunopathology, and control of bacterial replication as encapsulated caseous necrotic lesions (**Figure 2A**), granulocytic pneumonia, and cellular inflammatory lesions (Irwin et al., 2015). Unlike most humans, C3HeB/FeJ mice fail to control bacterial replication, especially in neutrophil predominant caseous necrotic lesions and pneumonia-like lesions, which may enclose more than 10^9^ bacilli, thereby causing high morbidity within 5 to 6 weeks after infection (Driver et al., 2012; Lanoix et al., 2015; Ordonez et al., 2016). In addition, C3HeB/FeJ mice can be stimulated to form cavities more frequently by administering incomplete chemotherapy with INH, RIF, and PZA (HRZ) (Irwin et al., 2015; Lanoix et al., 2015; Ordonez et al., 2016). Moreover, some of the animals developing cavities during follow-up remained clinically healthy for at least 2 months, suggesting the opportunity to develop a retreatment or cavitary TB model. Several anti-TB drugs have demonstrated differential treatment responses in C3HeB/FeJ mice compared to BALB/c mice which do not form caseous necrotic lesions (Driver et al., 2012; Irwin et al., 2014; Irwin et al., 2016; Lanoix et al., 2015). Clofazimine (CFZ) was highly effective in BALB/c mice, but its activity was attenuated in C3HeB/FeJ mice (Irwin et al., 2014). Moreover, treatment of C3HeB/FeJ mice with a single drug often displays heterogeneous responses. For example, PZA and linezolid (LZD) monotherapy was uniformly efficacious in BALB/c mice, whereas two distinct treatment outcomes were observed in C3HeB/FeJ mice: 1) responsive and 2) less responsive (Irwin et al., 2016; Lanoix et al., 2015). The discrepancy of treatment outcome is directly linked to the presence or absence of encapsulated caseous necrotic lesions in these mice (Lanoix et al., 2015; Irwin et al., 2014; Lanoix et al., 2016b; Irwin et al., 2016). Nevertheless, a relapse model has been developed with this mouse, and it has shown the sterilization and treatment-shortening effects of PZA given beyond two months (Lanoix et al., 2016a). C3HeB/FeJ mice have also demonstrated treatment-shortening with high dose rifapentine (RFP) and CFZ in combination with INH, PZA, and EMB (Saini et al., 2019). Non-invasive positron emission tomography (PET) coupled with computed tomography (CT) imaging has been extensively employed as a tool for monitoring disease progression as well as response to TB chemotherapy. Davis et al. (2009) demonstrated an evaluation of treatment responses of several TB regimens, including the first-line TB regimen of HRZ in C3HeB/FeJ mice using [18F]-2-floro-deoxy-D-glucose (FDG) PET/CT imaging. The study has revealed that PET/CT imaging correctly identified bactericidal activity of the drug regimens as compared with standard microbiologic methods and the application of non-invasive FDG PET/CT imaging in C3HeB/FeJ mice to monitor real-time treatment response as well as disease relapse. More recently, biodistribution of radiolabeled anti-tubercular drugs including INH, RIF, and LZD has been measured in various organs of C3HeB/FeJ mice following *Mtb* infection using PET/CT imaging, demonstrating an *in vivo* option for optimizing drug doses in treatment regimens (Weinstein et al., 2012; DeMarco et al., 2015; Mota et al., 2020).

C3HeB/FeJ mice are also utilized as a latent TB model. Dutta et al. (2014) described the development of latent model by immunization with a recombinant BCG 6 weeks prior to *Mtb* aerosol infection as described in BALB/c mice (T. Zhang et al., 2009), which resulted in a reasonably stable paucibacillary infection (4 log/lung). Antibiotic treatment at 6 weeks PI with INH or RIF for 2 to 4 months resulted in mice with very low bacterial loads (<1.5 log) that could be induced to relapse with tumor necrosis factor neutralizing antibody treatment. The potent activity of bedaquiline (BDQ), observed previously in BALB/c mice, was confirmed in this immunized C3HeB/FeJ latent model of TB (Lanoix et al., 2014). C3HeB/FeJ mice have proved a versatile murine model of TB to further understanding of TB pathogenesis and developing new therapies. However, the selection that occurs through the considerable early mortality, high bacterial loads, and intra-mouse heterogeneity of lung pathology within the same experiment, may be a potential limitation of C3HeB/FeJ mice (Irwin et al., 2015; Lanoix et al., 2015). The B6J.C3-Sst1 mouse has been suggested as possible improvement to using the C3HeB/FeJ mouse for both active and LTBI models in that it contains the sst1 locus conferring formation of necrotic lesions in the background of the B6 genetic resistance factors that slow disease progression, limit the bacteria to predominantly the necrotic core rather than the surrounding cellular regions of the granuloma, and lower bacterial loads (Kramnik and Beamer, 2016; Pichugin et al., 2009). As these mice become more available, investigators may explore this possibility as another approach to model human disease.

A humanized mouse model is commonly used for the study of TB co-morbidity with HIV (Sun et al., 2007; Marsden et al., 2012; Denton et al., 2012). The humanized mouse is generated when immunodeficient mice are reconstituted with human immune systems using various methods such as transgenic expression of human genes, adoptive transfer of human peripheral blood lymphocytes (PBLs), and human stem cell transplantation (Denton and Garcia, 2011; Ito et al., 2012). Calderon et al. developed a bone marrow, liver, thymus (BLT) humanized mouse with NOD/SCID *γ*c(null) mice by transplantation of human fetal liver and thymus-derived hematopoietic stem cells, creating a unique mouse model that retains the advantages of a mouse model with the immune system of humans (Calderon et al., 2013). BLT humanized mice infected with *Mtb* (10^6^ CFU) intranasally exhibit a progressive infection in the lung with dissemination to the spleen and liver from 2 to 8 weeks PI. These mice exhibit heterogeneous lung granulomas, including caseous necrotic lesions, as well as distribution of human T cells throughout the sites of inflammation (Calderon et al., 2013). A recent study demonstrated that two regimens, HRZ and HRZM (INH, RIF, PZA, MOX), reduced lung bacterial loads similarly in humanized mice and concluded the addition of MOX with the standard TB regimen had no additional benefits, confirming similar drug efficacy observed in human trials (Arrey et al., 2019). Despite the expense of humanized mice, they have significant advantages including the availability of human immunological reagents and mimicking human immune system (Calderon et al., 2013). After *Mtb* infection, humanized mice have more homogeneous bacterial loads and similar heterogeneity of lesion pathology resulting in a more consistent and reproducible mouse model (Arrey et al., 2019) when compared to the C3HeB/FeJ mice, (Irwin et al., 2015; Li et al., 2015).

Collaborative Cross (CC) mice are a panel of recombinant inbred mice that are derived from eight genetically diverse sets of founder mice, consisting of five inbred laboratory strains including: C57BL/C and A/J, as well as three wild-derived lines using a specific mating scheme (Svenson et al., 2012). These mice retain a large pool of genetic diversity as individual inbred lines, allowing genotypic reproducibility (Churchill et al., 2004; Collaborative Cross, 2012). CC mice infected with *Mtb via* aerosol (50 to 200 CFU) exhibit a broad range of susceptibility and lesion heterogeneity between strains. Resistant strains display lymphocyte aggregates, no necrosis, and fewer neutrophils in the lungs, whereas, susceptible strains display neutrophilic inflammatory lesions and widespread necrosis in **the lungs (** Smith et al., 2016). The CC mice have demonstrated the role of host genetic diversity in determining TB vaccine efficacy and elucidated the basis of TB susceptibility (Smith et al., 2016). Although the response to anti-TB chemotherapy has not yet been characterized in this model, it may prove to be a valuable resource for TB drug development and HDT testing, especially if the function of the compound is host microenvironment dependent.

### Outbred Models

Outbred mice are bred to maximize genetic diversity and heterozygosity within a population. Created by a rotational breeding scheme to prevent inbreeding, these mice are usually heartier and larger than inbred mice (Chia et al., 2005; Tuttle et al., 2018). Most early studies in TB drug development have been performed with outbred strains such as ICR and Swiss Webster (McCune et al., 1956; Grumbach et al., 1964; Grumbach, 1965; Grumbach et al., 1967; Grosset, 1978; Mitchison, 1980). The main advantages of outbred mice are their incomparable genetic diversity and low cost. However, there are some disadvantages of outbred mice as an experimental model: a large number of animals are required to maintain statistical power due to genetic diversity, and they are “genetically undefined”, meaning the genotype of an individual is unknown (Olson and Graham, 2014). Diversity Outbred (DO) mice are a new outbred mouse population derived from the same eight founder strains as the Collaborative Cross (CC) inbred strains (Collaborative Cross, 2012; Svenson et al., 2012) and retain the same set of genetic variants as CC mice (Churchill et al., 2004; Svenson et al., 2012; Smith et al., 2016). Niazi et al. (2015) described wide heterogeneity of responses in DO mice after low-dose *Mtb* infection *via* aerosol. DO mice exhibited three classes of response to *Mtb* infection based on survival, morbidity, and lung lesions at 5 weeks PI: super-susceptible, susceptible, and resistant. Recently, DO mice have been utilized for vaccine studies against TB and exhibited heterogeneous protection from *Mtb* infection, better modeling the diverse spectrum of outcomes in humans (Kurtz et al., 2020). Non-reproducibility of DO mice as a unique individual may be mitigated by parallel studies with inbred CC mice (Smith et al., 2016). To date, there has not been an anti-TB drug assessment reported in DO mice, but these mice may be another valuable resource for TB drug development when heterogeneity of response is desired.

### Persistence Models

Latent TB refers to a clinically asymptomatic stage of infection with the presence of antigen-specific T cell responses (Barry et al., 2009). Lung lesions in individuals with latent TB have been characterized as having minimal inflammation, prominent sclerosis, fibrosis, and calcification, but PET/CT imaging has provided additional insights suggesting that these lesions are not as static as previously thought (Lenaerts et al., 2015; Esmail et al., 2014). The Cornell model is a historical mouse model of latent TB (McCune et al., 1956; McCune and Tompsett, 1956). In this model, mice are infected with a high dose of *Mtb* (1 to 3 × 10^6^ CFU) intravenously and then treated with anti-tubercular drugs (INH and PZA) for 12 weeks. As a result, no viable bacilli are detected in the organs, but 90 days after termination of the drug treatment, reactivation of infection can occur spontaneously or by host immunosuppression (McCune et al., 1966b; McCune et al., 1966a; McCune et al., 1956; Flynn and Chan, 2001). Ever since first described by McCune and colleagues in the 1950s, various modified Cornell models have been developed by optimizing various parameters such as: infection dose, drug regimens, treatment length, and the antibiotic-free rest period (Scanga et al., 1999). Efficacies of several RIF or RFP containing regimens for preventive chemotherapy were measured using the Cornell model (Dhillon et al., 1996; Miyazaki et al., 1999), in which combined treatment of RFP and INH showed efficacy for short-course preventive therapy (Miyazaki et al., 1999). This model is attractive in that mice have low bacilli burden, resembling LTBI in humans; however, it can lack stability and consistency as the outcome of Cornell model-based studies depends upon a myriad of experimental parameters that are used to establish the model (Scanga et al., 1999). Another approach to creating a paucibacillary and stable *Mtb* population for testing LTBI regimens was to aerosol vaccinate BALB/c mice with *Mb* BCG before *Mtb* infection, avoiding the need to induce LTBI with antibiotics (Nuermberger et al., 2004). Improvements in this model by substituting a more immunogenic BGC vaccine strain (rBCG30) lowered the bacterial load further; these models showed that the combination of RFP and INH was more rapidly sterilizing than INH alone (Nuermberger et al., 2005; Zhang et al., 2009). This shorter RFP/INH regimen was later validated in humans (Sterling et al., 2016). Nevertheless, the Cornell model has been extensively utilized, particularly for drug evaluation and attempts to locate the reservoir of *Mtb* resulting in relapse (Dhillon et al., 1996; Miyazaki et al., 1999; Das et al., 2013; Liu et al., 2018). Operating on the hypothesis that the bone marrow is the source of *Mtb* in relapse, a recent study described a novel strategy for *Mtb* clearance post-chemotherapy, using nanoparticle-encapsulated drugs to specifically target and kill bone marrow resident *Mtb* in the Cornell model (Garhyan et al., 2020).

The main advantages of mice as a model are: low cost, ease of handling, low space requirements, low compound requirements, the availability of well-characterized immunological reagents, and the ability to use inbred, outbred, and transgenic strains (Franzblau et al., 2012; Harper et al., 2012; Vandamme, 2014). In particular, their genetic tractability and small compound requirement make mice the most practical model for compound assessment in the early phases of drug development (Nuermberger, 2017). There is no doubt that mice have been instrumental in TB drug development, but mouse models can differ from human disease in important ways, particularly the bacillary burden can become very high in susceptible mice, whereas less susceptible mice may not reproduce the full spectrum of lesions. Other disadvantages include metabolic differences that make modeling human-like drug exposures difficult and differences in susceptibility to toxic compounds.

## Guinea Pigs: The Most Susceptible, Immune Competent Model

Dunkin–Hartley Guinea pigs (*Cavia porcellus*; GP) are extremely susceptible to developing tuberculosis with progressive disease and a range of lesion types, making them an attractive model for drug testing and vaccine studies (Clark et al., 2014; Martinez et al., 2019; Chan et al., 2020). These animals have been infected through aerosol generation, IV or IP injections, or instillation *via* the nares or trachea, although low-dose aerosols may be the most common route in recent experiments (**Table 1**). The time required to show weight loss and changes of feeding behavior was directly related to *Mtb* dose delivered and strain virulence; most *Mtb* strains tested have caused disease in GPs, even at very low bacterial doses (Be et al., 2011; Shang et al., 2011; Kato-Maeda et al., 2012; Bottai et al., 2020). Lesions were common in extrapulmonary sites with exacerbated disease also reflecting the virulence of the *Mtb* strain. Typically, a low dose (20 to 50 CFU) of *Mtb* was the target to seed into the lung. The chronic infection stage in the lungs occurs after ~3 weeks; control of the low-dose infection is lost at about 6 to 9 months. The lung pathology was characterized as a combination of non-necrotizing and necrotizing granulomas (**Figure 2B**) (Basaraba, 2008; Basaraba and Hunter, 2017). The necrotic lesions have resident bacteria in the acellular margin surrounded by a hypoxic cellular rim that was difficult to sterilize (Lenaerts et al., 2007; Via et al., 2008; Hoff et al., 2011). Cavities, however, have rarely been observed (Helke et al., 2006; Clark et al., 2014).

The *Mtb* infected guinea pig appears to have been the first *in vivo* animal model of streptomycin activity against TB (Feldman et al., 1947b; Feldman et al., 1947a). Drug evaluation in the low-dose experimental infections in GPs enjoyed a resurgence after the BALB/c and C57BL/6 mouse models’ lesion structure was pointed out to be importantly different than those of humans (Tsai et al., 2006; Lenaerts et al., 2007; Basaraba, 2008; Coleman et al., 2014a). GPs have been treated with varying doses of HRZ, occasionally including testing for relapse (Lenaerts et al., 2008; Ahmad et al., 2011b; Dutta et al., 2012). HRZ given at 10, 12, and 25 mg/kg respectively for 6 weeks resulted in about a 1.75 log reduction in CFU, while 15 mg/kg of TMC-207 (active compound in BDQ) resulted in about a 4 log reduction (Lenaerts et al., 2007). Two months of treatment with HRZ followed by 2 months of HR was found to sterilize GPs with no relapse for 4 subsequent months, and PZA was active in the GPs at doses above 150 mg/kg (Ahmad et al., 2011a; Ahmad et al., 2011b). In a higher dose infection (200 CFU) beginning treatment 6 weeks PI, 2 months of daily RIF (100 mg/kg), or RFP (100 mg/kg) in combination with INH (60 mg/kg) and PZA (300 mg/kg) were found to be equally effective in reducing lung bacterial burdens and preventing relapse in the lungs (Dutta et al., 2012). An alternative regimen, Pa-824/MOX/PZA (25 mg/kg twice daily (BID), 90 mg/kg BID and 300 mg/kg once daily (SID) respectively), was compared with HRZ (60 mg/kg, 100 mg/kg and 300 mg/kg SID respectively). All GPs were culture negative after 2 months of treatment, and there were no bacilli detected after a relapse period of 2 months post treatment cessation (Dutta et al., 2013a). More interesting was thioridazine that showed activity in mice but little dose-related activity in the GP, leading the authors to suppose that the drug was not active against the extracellular bacteria (Dutta et al., 2013b). In standard drug therapy experiments with new drugs including Delamanid (DLM or D), alone and in combination with RIF and PZA for 4 weeks, the hypoxic lesions were clearly absent in GP lungs treated with regimen DRZ, in which 100 mg/kg DLM replaced INH in the HRZ (Chen et al., 2017). These results strongly suggested that DLM has bactericidal activity against *Mtb* in hypoxic lesions and can speed up bacterial eradication. GPs have been used extensively in the testing of aerosol chemotherapy formulations, including dry powders and nanoparticle preparations, in part because they are somewhat amenable to multiple blood draws for pharmacokinetics/pharmacodynamic (PK/PD) studies (Hanif and Garcia-Contreras, 2012; Horváti et al., 2014; Montgomery et al., 2018).

HDTs in the *Mtb* guinea pig model have taken a number of approaches: from using drugs or compounds to inhibit known host pathways to adding therapeutic vaccines to chemotherapy. Tissue-targeting strategies have also been tested. Based on the observation that oxidative stress during *Mtb* infection could promote tissue damage, Palanisamy et al. (2011) treated GPs with the antioxidant drug N-acetyl cysteine (NAC). NAC treatment reduced necrosis of GP lesions in the lung and spleen and slowed dissemination of *Mtb* to the spleen. A MMP inhibitor doxycycline suppressed *Mtb*-dependent MMP-1 and -9 secretion from human macrophages and epithelial cells (Walker et al., 2012). In the guinea pig model, doxycycline reduced lung *Mtb* burden after 8 weeks in a dose-dependent manner compared with untreated animals. *Mtb* avoids death in the macrophage by preventing the acidification of the endosome after phagocytosis (Sturgill-Koszycki et al., 1994; Bach et al., 2008). As part of this evasion, *Mtb* produces factors that interact with host proteins to subvert cellular processes. As an example, *Mtb* promotes the activity of the host tyrosine kinases Src/Abl, leading to a reduction in phagosome maturation and autophagy; when a Sar inhibitor (AZD0530) was added to cell cultures or administered to GPs, it promoted phagosome acidification and reduced mycobacterial growth accompanied by a reduction of pathology when compared to untreated controls (Chandra et al., 2016). Targeting similar virulence features of *Mtb*, Dutta et al. (2016) constructed small molecule inhibitors of mycobacterial protein tyrosine phosphatases (PTPs). These PTP dephosphorylate macrophage proteins would normally lead to phagosome acidification and infected-cell apoptosis, thereby preventing bacterial killing. The inhibitors, by preventing the activity of the PTPs, allowed the macrophage to inhibit bacterial growth in cell culture and in the guinea pig (Dutta et al., 2016; Vickers et al., 2018). The therapeutic vaccination approach has treated GP tuberculosis with a range of vaccine products from a specific gene target to eradicate persistent *Mtb*, such as relA DNA or heat-killed organisms, as a more general immunotherapy in combination with chemotherapy (Chahar et al., 2018; Chuang et al., 2020).

Tuberculous meningitis has been studied in the GP to gauge both strain virulence and dissemination, but not extensively enough for the purposes of drug development (Brown and Clark, 1962; Be et al., 2011). Guinea pigs have also proven useful in the study of type 2 diabetes and tuberculosis. In GPs provided with sucrose–water concurrent with low-dose *Mtb* infection with H37Rv to generate hyperglycemia, there were more *Mtb* lesions in the spleen and draining lymph nodes 30 days PI (Podell et al., 2012). Sixty days PI, the sucrose GP group had more bacterial burden as well as lung and spleen lesion area when compared to the water only control group, but both groups had advanced glycation end products indicating the occurrence of hyperglycemia as a result of *Mtb* infection as well as diet. In a second experimental model, diabetes was primed using a diet composed of 30% fat and 40% carbohydrate (to cause glucose intolerance) (Podell et al., 2014; Podell et al., 2017). After 8 weeks of the high-fat, high-carbohydrate diet, induction of beta cell death was performed by injecting the *α*_2_-antagonist prior to injecting streptozotocin (STZ) causing frank diabetes. The diabetic GPs demonstrated altered lipid metabolism and reduced compensatory *β*-cell capacity, but they were responsive to antihyperglycemic therapy with 100% survival (Podell et al., 2017). GPs with uncontrolled diabetes prior to infection with *Mtb*, had higher *Mtb* bacterial burdens, more severe organ pathology and shortened survival, commiserate with the increased pathology observed (Podell et al., 2014). This model has the potential to elucidate the complex therapeutic needs of TB patients with diabetes by evaluating if a specific drug treatment strategy is efficacious in them, or if longer or more intensive treatment is needed.

Guinea pigs present many of the advantages of mice as a model system: relatively small size, ease of handling, low cost, as well as a short time-course of infection. Their high susceptibility to *Mtb* infection provides a model for studying variable or low virlence strains; however, their low rate of developing cavitary pathology may be a limitation to the translation of *Mtb* chemotherapy efficacy from this model to humans. One significant advanatage may be in modeling diabetes and TB in a host with hypoxic, complex pathology.

## Rabbits: The *Mtb* Resistant Model Useful for Differential Compound Distribution

**Figure 3:**
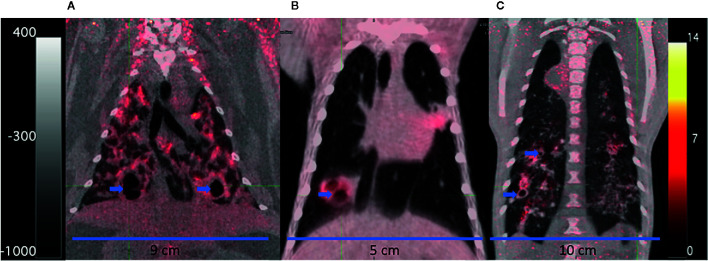
FDG PET/CTs of a rabbit **(A)**, marmoset **(B)**, and rhesus macaque **(C)** with cavitary disease. The animals were infected for 69 to 90 days with *M. tuberculosis* at the time of imaging. Cavities (blue arrows) have been partially emptied of their necrotic contents and filled with air indicated by a darker central region in the lesions (lower density) surrounded by lighter walls (higher density). The scales show the range of CT Hounsfield units from higher to low density in shades of gray (+400 to −1000) on the left and FDG uptake in PET standard uptake units/body weight from high to low uptake in bright yellow to red to black (14 to 0) on the right. The width of the animal’s midsection is indicated with a bar and label to highlight the difference in size of the three animals.

Rabbits (*Oryctolagus cuniculus*) are one of the most widely used non-rodents for studying human diseases, because although they require significant cage space, they are generally docile and easy to manage without sedation. Rabbits, including the New Zealand White (NZW) strain, are more susceptible to *Mb*, which can be rapidly fatal, than they are to most *Mtb* strains (Lurie, 1953; Dannenberg, 1994). The NZW rabbit can be infected with mycobacteria by IV, intrathecal, intrabronchial, or aerosol exposure. Lurie (Lurie, 1949, 1953) first described the range of pathological features rabbits shared with humans upon *Mb* exposure as ranging from: absence of evidence of disease to large caseous necrotic (**Figure 2C**) and cavitary pulmonary lesions (**Figure 3A**) that progressed within the lung by bronchogenic spread. With the currently available NZW rabbit, some *Mtb* Beijing (lineage 2) strains, including HN878, establish a chronic infection that includes the typical human range of disease: including necrotic pneumonia, hypoxic necrotic granulomas, liquefaction and cavitary disease that is bacterial dose dependent (**Table 1**) (Via et al., 2008; Tsenova et al., 2020). The bacterial load peaks 4 to 5 weeks PI, then most rabbits will develop chronic stable infection for 2 to 4 months. With prolonged infection, some rabbits will control the disease and have few residual paucibacillary lesions by 5 to 6 months; while others, with no outward signs of disease, develop large cavities (2–4 cm) containing ~10^6^ CFU/g caseum (Via et al., 2012; Sarathy et al., 2018). In contrast, aerosol exposure to *Mtb* strains H37Rv and hyperimmunogenic CDC1551 has been reported to result in latent disease with very low bacterial numbers after 10 to 12 weeks, which can be reactivated by immunosuppression (Manabe et al., 2008; Kesavan et al., 2009; Subbian et al., 2012). As a confirmation of the differential virulence of *Mtb* strains, Tsenova et al. (2020) explored a mixed infection model with HN878 and CDC1551 and found that hypervirulent HN878 persisted and grew in the lungs, while the CDC1551 was slowly cleared. In addition, the presence of CDC1551 in the lung had only a minor influence on the host’s immunological response to HN878, suggesting that immunologic control is local rather than systemic. To date, use of this model for testing sterilization rates of LTBI treatment regimens has not been published, but it appears that the rabbit would be appropriate for this modeling although housing space could be prohibitive.

The rabbit model has been used to address questions about toxicity, PK/PD, and efficacy of anti-tuberculosis drugs including INH, RIF, PZA, the PZA metabolite pyrazinoic acid (POA), and MOX among others (Via et al., 2012; Lanoix et al., 2016c; Blanc et al., 2018b; Sarathy et al., 2019). In studies designed to test the impact of hypoxia in *Mtb* lesions, metronidazole (MTZ) was shown to decrease the bacterial burden of NZW rabbits infected with *Mtb* HN878 in contrast to the lack of MTZ activity in C57BL/6 mice (Dhillon et al., 1998; Via et al., 2008). In longitudinal studies that combined PET/CT imaging, using FDG injected IV as a probe for metabolic activity, FDG concentrated in metabolically active regions within the *Mtb* HN878 lesions as they developed, but as necrosis progressed, the acellular centers of granulomas and cavities accumulated less FDG due to the lack of live cells (**Figure 3A**) (Via et al., 2012). TB lesions were observed to progress and resolve independently in the same lung without drug treatment, and individual lesions responded differently to different drugs. Chemotherapy with either INH or RIF during chronic infection reduced bacterial loads with quantitative changes in FDG uptake, lesion CT density, and total lesion volume. FDG uptake was reduced significantly with as little as 1 week of RIF treatment, indicating that FDG uptake might report more quickly on drug activity than other measures. Another PET probe, constructed from RIF labeled with [^11^C] has shown that within an individual, there can be spatially heterogeneous distribution of the probe and the drug in distinct TB lesions in both rabbits and humans (Ordonez et al., 2020).

Rabbits, with their large lungs that can host a large number of lesions without significant health effects, have proven to be highly useful in evaluating drug distribution, lesion penetration and cellular accumulation of drugs in different lesion types using laser microdissection and high sensitivity MALDI-MRM-MS imaging methods (Hudock et al., 2014; Sarathy and Dartois, 2020). Through these technologies, we have learned the location of the bacteria in the lesions though their lipid content and that MOX may preferentially accumulate in foamy macrophages (Blanc et al., 2018a; Blanc et al., 2018b). In a comparison of fluoroquinolones, Sarathy et al. (2019) showed that MOX was: more active than levofloxacin and gatifloxacin in the caseum *ex vivo* assay, was superior in sterilizing cellular and necrotic rabbit lesions during *in vivo* treatment, and was predicted to be more active in the *GranSim* model (Pienaar et al., 2017; Blanc et al., 2018b). In a similar study, both RIF and RFP had a ≥1 penetration coefficient in cellular lesions compared to plasma, but in cavities RIF penetrated at ~1, and RFP had only 0.25 penetration coefficient compared to plasma (Rifat et al., 2018). The lower penetration of RFP into the cavities is thought to explain why RFP required four times higher dosing than the standard to improve outcomes in patients with big cavities as compared with patients without cavities. In addition, these studies may have solved the mystery of EMB’s contribution to the INH, RIF, PZA, and EMB (HRZE) regimens. EMB was shown to have sustained accumulation above the MIC in both cellular and necrotic lesions; likewise PZA’s high penetration potential may promote its slow but potent activity in necrotic lesions (Zimmerman et al., 2017; Blanc et al., 2018c). Studies of *ex vivo* caseum have at least partially explained the phenomena of phenotypic drug tolerance through showing that *Mtb* bacilli native to cavity caseum are generally non-replicating and extremely drug tolerant (Sarathy et al., 2018). Only the rifamycin’s demonstrate sterilizing activity in this unique *ex vivo* assay that contributes a new tool to the characterization of drug activity against slowly or non-replicating bacteria. Taken together, these studies indicate that PK/PD data from the site of infection is needed to predict drug efficacy, and that similar characterization of new compounds is necessary when choosing among similar molecules to push further into late stage development.

As tuberculosis can occur in most organs of the body, there are specialized models for some of the more common and difficult to cure extrapulmonary sites of infection. Skeletal tuberculosis is still a common occurrence in endemic countries, and it is commonly treated with extended HRZE; but as the bone is prone to abscesses, requiring surgery and debridement, and local drug concentrations are expected to be low, a number of groups have worked on implantable scaffolds containing INH and/or RIF in the NZW rabbit model of bone tuberculosis (X. Huang et al., 2016; Li D. et al., 2017). Tubercular meningitis (TBM) occurs in 1–2% of TB cases and is one of the most severe forms of TB, especially among children, so several investigators have developed adult and pediatric TBM rabbit models (Tsenova et al., 1998; Tucker et al., 2016; Jain et al., 2018; Sánchez-Garibay et al., 2018). Recently, it was determined that DLM concentrations in the brain of naïve and TBM rabbits were five-fold higher than in plasma at both the maximum and trough concentrations, although the concentration in the cerebrospinal fluid was much lower (Tucker et al., 2018). This finding was corroborated by all patients with TBM receiving DLM experiencing clinical improvement. Use of the [^11^C]rifampin PET probe in TBM rabbits demonstrated that RIF penetration to the brain was limited, but that like DLM, RIF concentrations were higher than the measurements in the CSF might have indicated (Tucker et al., 2018). PK modeling using the PET scan data of TBM rabbits and humans and *postmortem* mass spectrometry of the rabbit brain were used to predict that the RIF dose necessary to reach therapeutic levels in the brain was two to three times higher than the currently recommended pediatric dose. Often TBM treatment includes adjunctive corticosteroids added to standard therapy, but this original HDT is both toxic and only partially successful. Tsenova et al. (1998) used thalidomide to block the production of tumor necrosis factor alpha (TNFα) in combination with INH and RIF to reduce inflammation and prolong the survival of rabbits with TBM. While this approach was successful, thalidomide’s toxicity also stimulated a search for other TNFα blockers. Etanercept treatment aggravated lung pathology and caused bacteria loads in the lesions to increase, compared with infected untreated animals (Tsenova et al., 2014). The search for more selective inhibitors led to the development of phosphodiesterase-4 inhibitors. One of these compounds, CC-11050, significantly reduced inflammation and partnered well with anti-TB drugs (Kumar et al., 2019), leading to a phase 2 HDT clinical trial that evaluated CC-11050 for safety and preliminary efficacy with rifabutin-modified standard therapy in adult pulmonary TB (https://clinicaltrials.gov/ct2/show/NCT02968927). HDT approaches to spinal TB and pulmonary TB are also under study (Datta et al., 2015; Wang et al., 2018).

One of the advantages of the NZW rabbit is their natural resistance to *Mtb*; rabbits usually have asymptomatic disease once infected and remain healthy (without evidence of clinical signs of pain, distress, and/or discomfort) even with cavitary disease. They can be serially bled for PK/PD studies and orally dosed without sedation. This ability to measure PK/PD in the same animal that is infected and treated with a compound is highly important in that compound absorption and metabolism vary from individual to individual and may be important to understanding the antibacterial activity within the individual. The use of medical imaging has reduced the number of animals needed per group, since with a baseline image, composing groups of equal disease burden with fewer rabbits is feasible as is preforming an image-guided necropsy. Once an experiment has reached its end point, the lesions in the large lung of the rabbit can be dissected and divided for bacterial load, histology, and other assays to characterize the nature of the infection. Administering multiple aerosol doses of *Mtb* to pre-sensitized rabbits may produce a higher rate of cavities earlier PI for drug studies of cavitary clearance (Kübler et al., 2015). The disadvantages: as large animals, NZW rabbits are expensive to maintain, require significant space, and almost have as much drug product or investigational compound as macaque models. Thus, rabbits make a good choice for a second model of efficacy where complex pathology is required or for the specialized studies of drug/compound distribution or TBM and HDT described above.

## Non-Human Primates Make Modeling Complex Disease States Similar to Human Disease Possible

Both wild and captive non-human primates (NHPs) are susceptible to *Mtbc* when exposed; thus it is not surprising that there is a long history of using them to study tuberculosis chemotherapy (Schmidt, 1956; Prier et al., 1964; Schmidt, 1969; Sapolsky and Else, 1987; Michel and Huchzermeyer, 1998), although they are more frequently used for vaccine studies and basic immunology investigations. Several primates have been used as models for human tuberculosis including the rhesus macaque (*Macaca mulatta*, RM), the cynomolgus macaque (*Macaca fascicularis*, CM), and more recently the common marmoset (*Callithrix jacchus*). The NHPs are more physiologically and immunologically similar to humans (Mothé et al., 2015), and individuals are more genetically diverse than the previously discussed models, suggesting these species may better represent the diverse outcomes resulting from *Mtb* infection as seen in humans (Capuano et al., 2003; Flynn et al., 2003). Numerous cross-reactive immunological reagents are available to identify the cell types that compose the granulomatous response to *Mtb* infection (Brok et al., 2001; Nelson and Loveday, 2014; Flynn et al., 2015). The NHPs develop the full range of human-like lesions, although the extent of disease is seldom as severe as seen in humans; for ethical reasons, as NHPs develop signs of disease including cachexia, dehydration, and mood alteration they must be treated or euthanized. The spectrum of lung disease observed in NHPs and humans includes: tuberculous pneumonia, necrotizing lesions surrounded with a fibrotic cuff (**Figures 2D–F**), cavities (**Figures 3B, C**), and solid cellular lesions (Capuano et al., 2003; Lin et al., 2013; Via et al., 2013). In addition to closely resembling human pathology, the NHP models are amenable to most diagnostic and therapeutic methods used in clinical studies, such as medical imaging (**Figures 3B, C**), serial blood sampling, and bronchoalveolar lavage (BAL) sampling for pharmacokinetic monitoring within an individual (Lewinsohn et al., 2006; Lin et al., 2013; Via et al., 2013). As serial imaging is feasible, methods to follow both individual lesion development and regression PI have been developed (Via et al., 2013; White et al., 2017). Once an experiment has reached its end point, these larger animals yield rich sample sets for characterization of the nature of the infection. Sampling the extrapulmonary organs such as: axillary, thoracic, inguinal, and mesenteric lymph nodes; spleen; liver; and kidney, will often identify bacteria and small granulomas. Yet, setting up these studies in shared primate facilities can be challenging not only because of the animals’ larger space requirement and expense, but also because many animal ABSL-3 facilities are reluctant to house *Mtb*-infected NHPs because of the highly infectious nature of *Mtbc* to other NHPs.

### Rhesus Macaques and Cynomolgus Macaques

Some of the earliest reports of TB in NHPs were from suspected human transmission in zoos or during transport (Montali et al., 2001). Early studies were of tuberculin-hyperreactive treated with INH therapeutically to prevent worsening or transmission of disease and tuberculin negative rhesus macaques treated prophylactically (Schmidt, 1956). As early as 1953, researchers determined that some *Mtb* strains were more pathogenic in RMs than in others, and that pathology decreased in RMs with lower bacterial doses instilled intrabronchially (Schmidt, 1972), but all doses (10 to 10,000 CFU) were capable of causing progressive disease. Aerosol exposure with <50 CFU caused slowly progressing active disease in RMs but with more discrete pulmonary tubercles (Barclay et al., 1970). Aerosol *vs* intrabronchial instillation of similar bacterial doses led to similar clinical outcomes and disease burden in Indian RMs, but the pathology in the lungs differed in distribution. Disease was found to be distributed throughout the lungs with aerosol exposure, while intrabronchial instillation led to extensive localized disease near the site of bacterial delivery (Sibley et al., 2016). Walsh et al. (1996) first established the CM as a model of TB in 1996 using *Mtb* strain Erdman, again demonstrating that disease severity was directly proportional to the intratracheally delivered bacterial CFU. CMs exposed to >10,000 CFU universally developed rapidly progressive bilateral pneumonia and succumbed to the disease in <3 months. When exposed to <1,000 CFU, the CMs progressed slowly (mean survival rate of 5 months) with disease generally local to the instillation site. Low doses of bacilli (<100 CFU) could occasionally establish subclinical or possibly latent disease in these animals. In order to establish a more human-like proportion of asymptomatic or LTBI CMs, Capuano et al. (2003) lowered the dose of *Mtb* Erdman to ~25 CFU instilled by bronchoscope directly into the lower lung, resulting in ~60% of animals developing progressive disease and 40% remaining healthy and asymptomatic for over 1 year, thus establishing the asymptomatic model in the CM. Additional studies directly compared the types of macaques; by *Mtb* aerosol infection and utilizing either *ex vivo* MRI or *in vivo* PET/CT, it was verified that CMs are more resistant to developing active disease than RMs (Sharpe et al., 2009; Sharpe et al., 2016; Maiello et al., 2018). The exception is the Mauritian cynomolgus macaque; perhaps because of their greater genetic homogeneity due to founder effect, they have shown higher, more RM-like susceptibility to *Mtb* with less variability in pulmonary disease burden and gross pathology and higher monocyte to lymphocyte ratios than similarly infected RMs and Chinese origin CMs (Sharpe et al., 2017; Maiello et al., 2018; Sibley et al., 2019). The majority of studies have used *Mtb* strain Erdman as the infectious agent, but several studies have used the laboratory strain H37Rv, which also shows a clear dose to lesion number response from 7 to 1,000 CFU in RMs (Schmidt, 1972; Gormus et al., 2004; Lewinsohn et al., 2006; Zhang et al., 2011; Min et al., 2013; Rayner et al., 2013). These experimental infections resulted in varying levels of active *vs* subclinical disease, and lower infectious doses did not always result in consistent disease burden from laboratory site to site. These inconsistences led to the hypotheses that Chinese RMs are more susceptible to developing active disease than Indian RMs and/or that the *Mtb* H37Rv strain could be different from site to site (Ioerger et al., 2010). In contrast, RMs infected with *Mtb* strain CDC1551 generally developed asymptomatic disease in the absence of co-infection or immunosuppression (Mehra et al., 2011; Luo et al., 2014; Foreman et al., 2020). In summary, these NHP studies can be manipulated to develop asymptomatic to rapidly fulminate disease as needed. Investigators can fine-tune the disease presentation and rate of disease progression to suit the needs of their hypothesis by carefully choosing the NHP species, route of infection, *Mtb* strain, and dose. Even a RM neonatal model, through very low-dose *Mtb* Erdman aerosol exposure or intrabronchial instillation, has been developed (Cepeda et al., 2013). The infected infant RM developed Ghon complex-like lesions at 6 weeks PI, and histopathologic observations were consistent with early TB, but anti-TB chemotherapy has not yet been explored.

INH chemotherapy studies in NHPs began almost as early as the studies in humans, often for the purposes of saving valuable animals as much as for understanding drug action or toxicity (Schmidt et al., 1953; Schmidt, 1956). There was a long lapse in drug efficacy studies as investigations shifted to other models for reasons of both expense and ethics. The shift back to the NHP model began as the focus on granuloma structure and drug distribution differences among the different TB animal models gained attention (Aly et al., 2006; Tsai et al., 2006; Via et al., 2008). One approach to standardization has been to develop pathology scoring systems (Capuano et al., 2003; Sharpe et al., 2009). With the availability of serial medical imaging and the development of potential radiologic biomarkers for evaluating disease changes, treatment groups could include smaller numbers of animals and still demonstrate a treatment effect (Lewinsohn et al., 2006; Lin et al., 2013; Via et al., 2013; Sharpe et al., 2018; Gordaliza et al., 2019). As in the rabbit, PET/CT studies revealed that CM TB granulomas progress and resolve independently in the same lung; untreated animals often have resolving sterile lesions, and individual lesions respond differently to different drugs (Lin et al., 2013; Coleman et al., 2014a). Furthermore, the necropsy findings correlated with the overall PET/CT results, supporting their use as prognostic markers of active drug therapies for actively diseased CMs. FDG uptake in HRZE or RIF treated CM lesions significantly decreased with time, while INH treated lesions had a more mixed response. When the lesions were segregated by type, the drug-treated fibrotic, healing lesions were more likely to have reduced FDG uptake over the course of treatment. When *Mtb* infected CMs were treated with either linezolid (at a humanized dose) or AZD5847, a second-generation oxazolidinone (OXA), the bacterial burden in lesions was reduced, as was the percent of lesions with culturable bacteria. The total FDG uptake (or total glycolytic activity) of the OXA treated CMs decreased with treatment, and when compared with humans treated with linezolid and receiving FDG PET/CT scans, the magnitude of treatment response was similar, demonstrating the value of the model (Coleman et al., 2014a). In addition to the small molecule drugs tested above, a single-stranded oligonucleotide Aptamer was selected to bind mannose-capped lipoarabinomannan (ManLAM) on the surface of *Mtb* (Pan et al., 2014). The aptamer also strongly bound to the host cells’ mannose receptors, so it was tested for activity in *Mtb* infected mice and a small number of Chinese RMs. The aptamer treated RMs had ~50% lower bacterial burdens than the untreated animals and less evidence of granulomatous inflammation in the lung, suggesting it was able to reduce disease progression. The mechanism of action was suggested to be the reduction in ManLAM-induced immune suppression and enhancement of the antigen-presenting activity of dendritic cells. More work is needed as the sample size was limited, but the authors proposed this molecule could be developed as a new anti-TB agent or used as an adjuvant.

Additional TB drug studies have specifically targeted treatment of asymptomatic TB. In one study, the hypoxia-active drug MTZ was tested for activity in CMs confirmed to have asymptomatic TB 6 months after exposure to ~25 CFU of *Mtb* Erdman (Lin et al., 2012). MTZ was as effective as 2 months of INH/RIF or the standard 6 months of INH in preventing reactivation of TB through anti-TNF neutralizing antibody administration. MTZ paired with INH/RIF for 2 months also significantly reduced the bacterial burden in CMs with active TB, demonstrating that compounds with activity under hypoxic conditions could have a useful place in treating both active and latent TB. In a second study of persistent asymptomatic TB, Indian RMs were aerosol exposed to ~10 CFU *Mtb* CDC1551 and confirmed to have converted to mycobacteria-exposure positive (Foreman et al., 2020). Those RMs asymptomatic at 3.5 months PI were randomized to either receive treatment with INH and RFP (15 mg/kg each, once weekly for 12 weeks in diet) or left untreated. After 7 months, both groups of asymptomatic RMs were reactivated by simian immunodeficiency virus (SIV) coinfection (Mehra et al., 2011). None of the INH and RFP treated RMs developed signs of active infection within 3 months compared with 85% of the untreated RMs, suggesting the treatment prevented reactivation of asymptomatic RMs. Few recent investigations delve into TB drug activity in SIV/TB co-infected macaques, although macaques are used in modeling of HIV drug pharmacokinetics, drug interactions and efficacy, so the co-infection model will not be discussed further.

Finally, HDT administered in combination with anti-TB drugs is thought to be one approach toward achieving a shorter, more durable cure. In CMs, Winchell et al. (2020) targeted modulation of IL-1. IL-1 can contribute to inflammation and exacerbate pathology, which could reduce the activity of anti-TB compounds through limiting access to the bacilli. In addition, linezolid treatment may contribute to IL-1 promoted inflammation, so reducing IL-1 could produce more rapid bacterial killing by LZD. CMs were infected with ~12 CFU of *Mtb* Erdman and allowed to develop active disease for an average of 4 months, then randomly assigned to a group to receive either LZD (30 mg/kg twice daily) or LZD, and the IL-1 receptor antagonist. The two treatment groups had similar decreases in bacterial loads at the end of 4 weeks of treatment, but the combination treatment also had significantly reduced lung inflammation, suggesting that the combination could reduce patients’ residual lung pathology at the end of TB treatment, thereby improving overall health outcomes.

The advantages of the macaque models include: the excellent availability of reagents, similar PK/PD to humans, and availability of well characterized *Mtb* strains mentioned above from BEI Resources (Rockville, MD), including vials of a bar-coded *Mtb* Erdman library (Martin et al., 2017) available for direct use from BEI. The majority of macaques infected with *Mtb* develop a wide spectrum of human-like granulomatous lung disease. Those that developed LTBI developed one to several lesions within 3 weeks PI and then stable or regressive pathology (Coleman et al., 2014b). It is important to understand that lower-dose infections mimicking what is thought to be human-like exposure can take several months to develop a significant disease and as much as 6 months to confirm asymptomatic disease, so the time commitment for these studies is extensive. Another disadvantage is that natural infection occurs, so the absence of previous exposure to mycobacterial antigens should be confirmed by screening using an IFN-*γ* release assay or other method of screening and baseline lung imaging to rule out ongoing infection as recent work has confirmed that current infection with *Mtb* significantly protects CMs from a secondary *Mtb* infection (Cadena et al., 2018).

### Common Marmosets: The Compound-Sparing Model of Active Disease

The common marmoset (*Callithrix jacchus*) model of tuberculosis was developed specifically to support drug development efforts because of their small size, relative ease of handling and breeding, pharmacologic similarity to humans for drug development, and propensity toward fraternal twins and triplets (Via et al., 2013). In addition, disease progression and treatment response in marmosets were easily monitored through the use of anesthesia and serial medical imaging protocols (Hart et al., 2004). In tuberculosis studies that combined PET/CT imaging with FDG injected intravenously as the probe, FDG concentrated in metabolically active cells within the granulomas as they developed. Later at necropsy, all lesions were identified by using the PET/CT image as a guide, excised, and sampled for histology and CFU. Marmosets were found to be highly susceptible to aerosol and intratracheal *Mtb* infection with the rate of disease progression and weight loss correlating to both virulence of the *Mtb* strain and the infectious dose (Via et al., 2013; Cadena et al., 2016). Infection with either a Beijing clade strain or *Mtb* strain Erdman led to a rapidly progressive disease with either coalescing necrotizing granulomas with lymphocytic cuffs or tuberculous pneumonia with a neutrophilic infiltrate, respectively. With *Mtb* strain CDC1551 aerosol infection with 15 to 25 bacteria, all marmosets developed primary progressive disease. In addition, the majority developed a wide spectrum of lung granulomatous lesions: solid cellular lesions, necrotizing lesions surrounded with a fibrotic cuff (**Figure 2D**), tuberculous pneumonia, and cavitary lesions by 6 to 7 weeks before progressing to the humane endpoint at about 10 weeks post-infection (Via et al., 2013). If a very low bacterial dose (1 CFU) was presented, then survival was extended to >300 days in one marmoset, suggesting the possibility of developing a chronic model in this NHP. While small, the marmoset is large enough for intra-animal blood sampling to determine the pharmacokinetic profile of a compound in both naïve and *Mtb* infected individuals (Via et al., 2015). In addition, the affected organs, primarily the lung, are large enough to dissect into individual lesions and regions of apparently normal tissue, all of which can yield information about histopathology, bacterial burden and/or biochemical or compound distribution in lesions (Beites et al., 2019).

To benchmark the *Mtb*-infected marmoset response to treatment, two drug regimens known to differ in their relapse rates in human clinical trials were tested: the standard four-drug combination of HRZE that has a very low relapse rate and the combination of INH and streptomycin that has been associated with higher relapse rates (Fox et al., 1999). The results suggested that the basis for improved sterilizing activity of the four-drug combination was both its faster disease volume resolution and its stronger sterilizing effect on cavitary lesions (Via et al., 2015). The compound effects on bacterial load have been assessed after 4, 6, and 8 weeks of treatment thus far, with approximately 4 log reduction compared to controls observed with HRZE, similar to other models (Via et al., 2015). Treatment of *Mtb*-infected marmosets with a weak active inhibitor of the *Mtb* cytochrome bc1-aa3 oxidase stalled disease progression and weight loss, slightly reduced bacterial loads, and reduced lesion-associated inflammation as measured by FDG uptake; however, most lesions became cavitary by 2 months (Beites et al., 2019). The measurable endpoints in this model include the typical ones: survival, changes in body weight, organ bacterial burden, and histology of residual disease at the end of treatment. Also measurable are: changes in lung disease volume and inflammation through serial PET/CT imaging, compound exposure through serial blood draws, blood chemistry changes, lesion specific bacterial burden, compound concentration and compartmentalization, and image analysis of lesion-specific treatment response to compounds and regimens all within a five month experimental protocol (Beites et al., 2019).

The advantages of the marmoset model include: 1) the relative speed in which compound activity in complex lesions like cavities and other lesion types can be determined, 2) lower space requirements and costs than the RM or CM models, 3) human-like PK/PD, and 4) lungs large enough to divide into many individual lesions allowing for a larger intra-individual sample set than can be achieved with the mouse models. In addition, while marmosets are heterogeneous, breeding pairs frequently produce twins and triplets, allowing for some homogeneity across compounds during studies. The disadvantages are expense, complexity of housing and breeding, or scarcity if buying the animals, and fewer immunological reagents than macaques.

## Other *In-Vivo* Models Intended for TB Drug Development

In the search for an ideal TB model, investigators continue to test other animal species as models for TB and TB chemotherapy including various rats, the minipig, and the Chinese tree shrew among others (Sugawara et al., 2004; Elwood et al., 2007; Gaonkar et al., 2010; Gil et al., 2010; Zhan et al., 2014). Of these, the Wistar rat is perhaps the most well characterized, because it is a common species used for PK/PD characterization and toxicity assessment of potential therapeutics, and it would be more economical to be able to use the same species for drug efficacy studies (Gaonkar et al., 2010; Singhal et al., 2011; Foo et al., 2011; Ramani et al., 2012; Kondreddi et al., 2013; Kumar et al., 2014). The Wistar rat and Lewis rats lacked lesions with central necrosis when infected with the *Mtb* strains chosen by the investigators, but the two species of Cotton rat developed necrotic lesions and higher bacterial loads despite being exposed to a low dose of *Mtb* H37Rv (Sugawara et al., 2004; Swaim et al., 2006; Elwood et al., 2007; Singhal et al., 2011). Differential virulence of *Mtb* strains was observed in Wistar rats as judged by bacterial loads developed in the lung and rate of clearance, but rats exposed to a low-dose infection eventually began clearing even *Mtb* HN878, suggesting this rat could be developed into a LTBI model. Investigators exploring alternative methods for drug delivery, including respirable microspheres loaded with RIF or other anti-TB compounds, also often utilize the rat model (Hirota et al., 2013; Mustafa et al., 2016; Maeda et al., 2019). Infant, prepubescent, and adult minipigs have been infected with *Mtb* strains and explored as a model of TB and therapeutic treatment (Gil et al., 2010; Ramos et al., 2017). There is some indication that the minipig, like the rabbit, is resistant to developing severe disease unless infected with a virulent *Mtb* strain; the model has also been useful in constructing a safety profile for the iron-containing INH analog IQG-607 to support its clinical development (Rodrigues-Junior et al., 2017). In addition, this search has brought attention to the Chinese tree shrew, a close relative of primates, that weighs only 130–140 g. The shrew develops caseous necrotic lesions in the lungs and numerous other organ systems five to eleven weeks after IV injection with 10^6^ CFU of the *Mtb* strain H37Rv. After IV injection with 10^3^ CFU *Mtb* strain H37Rv, no gross disease or lesions were observed, but some tissue samples were culture positive, leading to the suggestion that the shrew could be used for LTBI studies (Zhan et al., 2014). It remains to be seen if these models will be developed further.

## Summary and Concluding Remarks

No one animal model has reproduced the human presentation of TB with both the genetic heterogeneity, age-related variations, and the many co-morbidities that human TB patients exhibit when they come to the clinic (Borgdorff et al., 2000; Workneh et al., 2017; Seddon et al., 2018). The main risk factor for relapse in human clinical trials is the presence of cavitary lesions, and these lesions are thought to be important in determining the length of treatment needed for a durable cure (Benator et al., 2002; Hamilton et al., 2008). Scientists continue to infect additional animal species with *Mtb* searching for a model that reflects as much of the spectrum of human tuberculosis as possible, in a small and easy to maintain animal. For now, the current goal should be combining the existing models efficiently with an eye not only to assessing the change in bacterial load, but also to identifying the host environments where the compound acts on the bacteria, and the residual pathology left behind once the bacteria are eradicated.

The zebrafish model seems more suited to compound screening for anti-mycobacterial activity than the larger animals in that the larva can live in 96 well plates for the duration of automated assays where bacterial load is measured by fluorescence (Takaki et al., 2013). These assays can deliver data similar to that of macrophage and *ex vivo* granulomas infection models particularly identifying compounds that may be active only in the presence of host cells (L. Huang et al., 2018). The adult fish are pigmented so more traditional methods of counting bacteria are necessary. In both cases, since *Mm* does not require a ABSL-3, early compound evaluations can be done in ABSL-2 laboratories. Mice are the workhorses of TB drug development. Initial PK/PD is usually determined in mice as are early determinations of efficacy in acute infections while the bacilli are still replicating (Nikonenko et al., 2004; Rullas et al., 2010). It is important to note that achieving the compound concentrations that are bactericidal in *in vitro* assays, *in vivo* is not enough to identify a preclinically or clinically useful dose (Gumbo et al., 2015a; Gumbo et al., 2015b). Protein binding for compounds which should be measured for the animal model that will be used as binding is often different among animal species. The methods for measuring drug concentrations in animals and their relevant tissues can be labor intensive, and in small animals where blood sample volume and collection frequency are limited, such as mice, it may require a large number of animals. Even though mice have very different metabolism than humans and the larger animal models, dose fractionation studies to determine which exposure parameters are most important for the activity of a compound are usually conducted in mice because they are inexpensive and require small amounts of compound—see Gumbo et al. (2015a) for a fuller description.

If compounds show activity in one of the more permissive acute mouse models, then the compound is tested in a more chronic mouse model after the immune response has developed (Gill et al., 2009; Hoff et al., 2011; Nuermberger, 2011). But at this point, only the intracellular activity of the compound has been assessed, so the choice of a model with extracellular bacteria populations becomes important as does the pathology the infection model develops. The C3HeB/FeJ mice, humanized mouse, GPs, rabbits, and NHP all develop populations of extracellular bacteria embedded in necrotic inflammatory cell debris within the lung both as regions of necrotic pneumonia and necrotic granulomas. The C3HeB/FeJ mice, rabbits, marmosets, and the two macaques develop cavities during infection and may have significant residual lung pathology at the conclusion of the therapy. One gap in our knowledge is just how similar the cavities in these models and human cavities are; another is if the strains we are using to generate these lesions represent enough of the *Mtb* heterogeneity for drug development purposes (Bottai et al., 2020).

Mouse lungs are usually processed for bacterial loads as a single sample, so differentiation of the bacterial load in specific pathological structures is not usually available, whereas this type of data is collected with rabbits, marmosets, and macaques allowing for estimates of lesion-specific bactericidal activity by pathologic assessment (Coleman et al., 2014a; Via et al., 2015; Sarathy et al., 2019). Recently, antibiotic therapy was shown to alter the number of some antigen-specific T-cells (Chuang et al., 2020), suggesting that there may be specific advantages to animal models like the rabbit that form cavities without requiring antibiotic treatment or vaccination to support survival. The inclusion of determining drug concentrations in both blood, naïve lung tissue, and lesion tissues is recommended as tissue penetration is often different among lesions and organs (Kjellsson et al., 2012; Prideaux et al., 2015; Ordonez et al., 2020). These studies are more easily accomplished in rabbits which have large lungs to host many lesions with few signs of clinical disease. Compound absorption and metabolism vary from individual to individual and can influence compound efficacy estimates. Sufficient blood sampling to estimate an individual animal’s compound exposure is ideal, thus using rabbit, marmoset, or macaques in late stage testing to estimate the effect of this variability is important as well as tissue collection for drug concentrations at the experimental endpoint.

Once a compound is ready to be combined with other drugs into possible regimens, a model of sterilization and relapse is usually employed. These were traditionally done in BALB/c mice, but recent efforts have developed a C3HeB/FeJ model that relapses in the same lung regions as earlier lesions (Murawski et al., 2014; Lanoix et al., 2016a). The macaques have also been used for detecting relapse using immunologic manipulation to speed relapse (Lin et al., 2012). Among the existing LTBI animal models, the Cornell model adaption in the BALB/c, C3HeB/FeJ mouse, and GP is treated with INH and other drugs to induce a state modeling LTBI. Aerosol vaccination of these mice with recombinant BCG also makes a reproducible model that produced sterilizing results encouragingly similar to those in humans (Nuermberger et al., 2005; Zhang et al., 2009; Dutta et al., 2014). As more of the human population is vaccinated with *Mb* BCG, these models may have even more relevance. In the more *Mtb* resistant models like the NZW rabbit and the macaques, LTBI can develop without treatment under specific infection conditions. The macaque LTBI models were shown to respond to the same LTBI treatments as humans (Lin et al., 2012; Foreman et al., 2020), but the rabbit LTBI model has not been similarly tested. These more resistant models deserve further development in order to standardize the models for future therapeutic testing.

Combination anti-TB drug treatment and HDT may be necessary to shorten treatment to 4 months or less and to reduce the residual lung pathology that remains at the end of treatment. One phosphodiesterase-4 inhibitor HDT that reduces pathology has shown promise in the rabbit and has moved into human trials (Subbian et al., 2016). Other HDTs have significantly reduced lung inflammation in CM (Winchell et al., 2020). Therapeutic vaccination as an HDT in combination with antibiotics was an effective approach to shorten TB treatment and/or reduce relapse rates in mice and guinea pigs (Chuang et al., 2020). Yet to be addressed are the complex questions related to why some human patients seem to be cured of *Mtb* and yet have remaining debilitating lung pathology (Malherbe et al., 2016; Lawal et al., 2020). Depending on the mechanism of action of these adjunctive therapies, some model species with greater similarity to humans may be more appropriate than others. For compounds with exceptional tissue distribution or that appear to cross the blood brain barrier, specialized bone TB and TBM models have been developed in GPs and rabbits. Two major co-morbidities of tuberculosis, HIV co-infection and diabetes have been developed and characterized in macaques and GP, respectively.

It is an exciting time in the effort to end tuberculosis. There are several new agents that have been approved for use in humans, several more in phase 2 clinical trials, and many researchers actively contributing compounds to the pipeline. To characterize these new agents, we must mindfully select additional *in vivo* tools that provide lesion-specific activity and compound exposure and distribution data to support their further development including support for composing regimens predicted to achieve the most rapid cure. And finally, the heterogeneity of both humans and the *Mtb* strains they are exposed to means that modeling TB requires models of multiple sizes and styles.

## Author Contributions

H-JY, DW, XW, DMW, and LV wrote the manuscript. DMW edited the draft manuscript. All authors contributed to the article and approved the submitted version.

## Funding

This work was supported in part by the intramural research program of NIAID and in part by the Bill and Melinda Gates Foundation (OPP1162695 to Clifton E. Barry 3^rd^). DW is the recipient of NSFC grants 31772709 and 31572485.

## Conflict of Interest

The authors declare that the research was conducted in the absence of any commercial or financial relationships that could be construed as a potential conflict of interest.
